# Antimicrobial Use and Resistance in Agriculture and Food Production Systems in Africa: A Systematic Review

**DOI:** 10.3390/antibiotics10080976

**Published:** 2021-08-13

**Authors:** Stephen E. Mshana, Calvin Sindato, Mecky I. Matee, Leonard E. G. Mboera

**Affiliations:** 1 SACIDS Foundation for One Health, Sokoine University of Agriculture, Morogoro 65125, Tanzania; mshana72@bugando.ac.tz (S.E.M.); csindato@gmail.com (C.S.); mateemecky@yahoo.com (M.I.M.); 2 Catholic University of Health and Allied Sciences, P.O. Box 1424, Mwanza 33109, Tanzania; 3 National Institute for Medical Research, P.O. Box 482, Tabora 45026, Tanzania; 4 Muhimbili University of Health and Allied Sciences, P.O. Box 65001, Dar es Salaam 11103, Tanzania

**Keywords:** antimicrobial, use, resistance, agriculture, food production, Africa

## Abstract

In Africa, there is dearth of information on antimicrobial use (AMU) in agriculture and food production systems and its consequential resistance in pathogens that affect animal, human and environmental health. Data published between 1980 and 2021 on the magnitude of AMU and AMR in agriculture and food productions systems in Africa were reviewed. Data from 13–27 countries in Africa indicate that 3558–4279 tonnes of antimicrobials were used in animals from 2015 to 2019. Tetracyclines and polypeptides contributed the largest proportion of antimicrobials used. Cattle and poultry production account for the largest consumption of antimicrobials in Africa. Although limited studies have reported AMR in crops, fish and beekeeping, AMR from a variety of farm animals has been substantially documented in Africa. Some countries in Africa have developed policies/plans to address AMU and AMR in agriculture and food production systems; however, their enforcement is challenged by weak regulations. In conclusion, although there is limited information on the quantities of antimicrobials used in agriculture and food production system, the levels of AMR are high. There is a need to strengthen regulatory authorities with a capacity to monitor AMU in agriculture and food production systems in Africa.

## 1. Introduction

Increases in human populations in Africa have triggered an increase in demand for food from agricultural and animal products. According to United Nations reports, the African population is expected to rise from 1.3 billion in 2019 to 2.4 billion in 2050 [1]. The increase in population is in tandem with the demand for food products. Therefore, more agricultural and livestock products need to be produced to cater for economic, social and dietary demand [2]. Already, the production of meat in Africa has risen by 64% since 2000, due to demand for high-protein diets [3], consumer preferences and urbanization [4], as well as for export. The profitable food production has involved both intensive and extensive production. Intensive production has been widely practiced by both small- and large-scale producers, because it has been considered to be a more profitable way of ensuring animal welfare and a solution for food security worldwide [5]. On the other hand, intensive animal production has been implicated with the development and spread of zoonotic diseases, environmental change resulting from pressure on natural resources and change in livelihoods [4,5].

Consumption of quality and safe food from plants is of crucial importance for the wellbeing of humans and animals. During production, contamination from different sources such as microbial organisms, chemicals and drug residues may be encountered, especially if compliance with food safety and hygienic measures is not followed [6,7]. As such, consumers are exposed to the risk of consuming traces of antimicrobial residues and the drug resistant organisms [8,9,10]. The needs for antimicrobials in agricultural production, especially in treatments, of plant diseases are expected to rise due to increases in the demand of fruits and vegetables as a result of the growing human population [6,11,12].

In intensive agriculture and food production systems, antimicrobial use (AMU), intended to maintain animal health and productivity, is inevitable [3]. Of great concern are negative effects such as the development and spread of resistant pathogenic micro-organisms. The resistant organisms can spread directly through contaminated animals and their products, crops, soils and ground and surface water [13]. The transmission of resistant micro-organisms can also occur during harvesting, post-harvest handling and distribution due to lack of compliance to hygienic practices [10]. AMU in humans, animals and agriculture are implicated to provide the selection pressure of antimicrobial resistance (AMR) genes from the environment, thus increasing the AMR burden.

In Africa, the increase in AMU and AMR is contributed largely by weak regulations in plant, human and animal health practices [14], weak enforcement of available laws and regulations on AMU, weak surveillance systems for AMR and AMU in humans, animals and crops, a lack of policies and regulation regarding the disposal of antimicrobials and a lack of updated standard treatment guidelines for human and animal diseases. This review was carried out to provide data on the magnitude of AMU and AMR due to the existing policies in agriculture and food productions systems in Africa.

## 2. Materials and Methods

### 2.1. Search Strategy and Inclusion Criteria

PubMed, Science Direct, MEDLINE, LISTA, Web of Sciences, Scopus, African Journal Online and Google Scholar were searched for relevant English or French articles published between January 1980 and May 2021. Combinations of search terms used were: ‘antibiotic use’, ‘antimicrobial use’, ‘antimicrobial usage’, ‘antimicrobial resistance’, ‘antibiotic resistance’, ‘antimicrobial resistant’, ‘agriculture’, ‘food-producing animals’, ‘food animals’, animal husbandry’, ‘animal farming’, ‘domestic animal farming’, ‘farmed animals’, ‘crop protection’, ‘soil’ and ‘vegetables’. Others were specific food animal descriptors such as ‘poultry’, ‘chickens’, ‘pigs’, ‘swine’, ‘cattle’, ‘beef cattle’, ‘dairy cattle’, ‘fish’, and ‘bee’, specific countries by name, and the word ‘Africa’. The articles were scrutinized to extract information on antimicrobial use, the prevalence of AMR and availability of a surveillance system.

Furthermore, in order to establish the impact of the policies in Africa, we searched information on AMU and AMR covering a period from 2005 to 2020. In addition, publications from the Food and Agriculture Organization (FAO), World Health Organization (WHO), Office International des Epizooties (OIE) and Africa Centres for Disease Control and Prevention websites were searched and reviewed. In each document, information regarding antimicrobial uses, resistance and surveillance was extracted. In addition, more information regarding the authority and enforcement of laws was also extracted.

### 2.2. Data Extraction and Assessment of Articles

Data extraction was performed using a pre-designed database in Microsoft Excel 2013. Data were abstracted from each article into standardized data abstraction tables with the following variables of interest: country, animal species, bacteria species investigated, resistance genes detected, antimicrobial classes investigated, percentage multi-drug resistance (MDR), type of policies, year of approval and whether in use or not. The quality of article/policy document was assessed. A limited number of articles/policy document were available; therefore, we did not use stringent criteria to exclude the article/policy documents from this review.

## 3. Results

### 3.1. Search Results and Selected Studies

**Figure 1:**
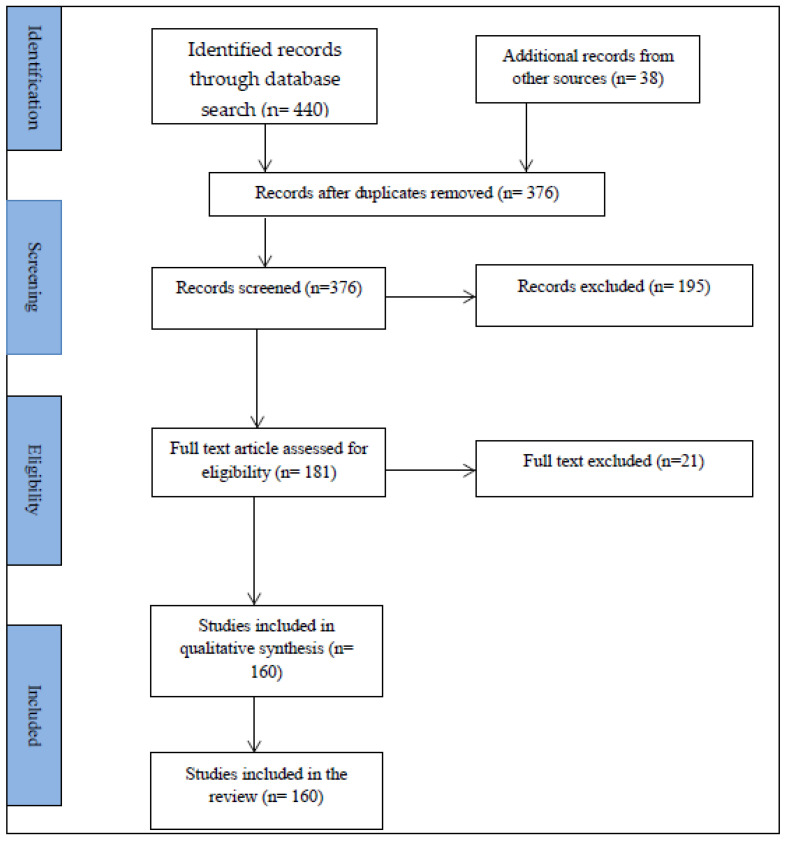
PRISMA flow diagram of the articles obtained, selected and included in the review.

A total of 440 and 38 records were retrieved from database searches and from other sources, respectively. After removing duplicates and screening, 160 studies were included in the final review (Figure 1).

### 3.2. Antimicrobial Use in Crop Production

This review found no study across the Africa continent that reported data on antimicrobial consumption in plants, types of antimicrobial used, and the quantity used. Thus, the amount of AMU in agriculture in Africa is not known with certainty. Globally, the estimated annual antimicrobial consumption in agriculture is more than 60,000 tonnes [12]. It has been difficult to estimate AMU in the agricultural sector due to inadequate data availability. Lack of data on AMU in agriculture in Africa is caused by several reasons, including the weak surveillance of AMU in agriculture, lack of, or weakness in the enforcement of regulations, and a lack of guidelines and standards for the surveillance of AMU and AMR [15].

Antimicrobials such as tetracycline, streptomycin and triazoles that are used to treat human and animal infections are also used to control plant diseases [8]; pesticides are used in the agriculture industry to optimize productivity. A study in Kenya, Uganda and Ethiopia reported up to a 47% loss in productivity if pesticides were not effectively used, and up to a 20% increase in productivity with the proper use of fungicides [16]. In Africa, contact fungicides such as Dithane M-25(mancozeb) and systemic fungicides such as Ridomil (metalaxyl), as well as benzimidazoles, are most frequently used [16]. Benzimidazole fungicides were extremely commonly used in the mid-20th century at quantities ranging from 1.5 to 3 kg per hectare and posed a very high risk of resistance development after 3–4 seasons of spraying [17,18]. Currently, triazoles, are the most commonly used fungicide, at a rate of 100 g/ha of plant surface due to their effectiveness and broad spectrum of activity [19]. In Tanzania, about 40 types of pesticides are used depending on their availability and accessibility to farmers; these include cyproconazole, mancozeb, copper oxychloride, mancozeb metalaxyl and propiconazole [20]. The usage of pesticides in Tanzania is reported to be at an extremely high level, whereby up to 50% of farmers apply a mixture of at least three pesticides during a single crop season depending on the types of crops [20,21].

### 3.3. Antimicrobials Use in Animal Production

AMU in animal production is of great concern; the trend shows the rise in consumption driven by increased demand for foods of animal origin. Globally, estimated antimicrobial consumption in the livestock sector in 2010 was 63,151 tonnes per year [22]. However, the availability of data on AMU in animal production in African countries is scarce [9]. Available statistics indicate that more than 75% of the African countries submitted AMU data, with 27, 28, 33 and 30 countries submitting quantitative data to the World Organization of Animal Health (OIE) in 2016, 2017, 2018 and 2020, respectively [23,24,25,26]. Data on AMU in aquatic animals were submitted from 7, 4, 15 and 8 African countries in 2016, 2017, 2018 and 2020, respectively [23,24,25,26]. Regarding animal biomass in Africa, cattle contributed 53% in 2017, with 48% and 48.6% in the 2018 and 2020, respectively. The contribution of poultry was 6% in 2017, 5% in the 2018 and 5.9% in 2020. The quantity of AMU in Africa adjusted by estimated coverage dropped from 4279 tonnes in 2017 to 3558 tonnes in 2020. Tetracycline contributed 63% of the quantity used in 2016, 11.6% in 2017, 31.7% in 2018 and 28.7% in 2020. Other common antimicrobials found to significantly contribute during the reporting period were macrolides, quinolones, penicillin and aminoglycosides [23,24,25,26].

Measures that are currently used are the defined daily dose animals (DDDA), course dose animal (DCDA), defined daily dose for veterinary medicines (DDDvet) and defined course dose veterinary medicines (DCDvet). The measures may vary from one country to another. A majority of the studies on AMU in Africa reported on qualitative rather than quantitative data [27]. However, of the few available studies that determined quantitative use through DDDA, a major limitation was that the data were not representative for the entire country or reported from a single animal species [28,29]. The number of African countries submitting AMU reports to OIE increased from 13 in 2014 to 20 in 2016, with respective percentages of total estimated biomass covered of 41%, 54%, and 51%. During the period, the quantities of reported adjusted estimated AMU were 4279, 3674 and 3558 tonnes in 2014, 2015 and 2016, respectively. It is important that African countries pay special attention to issues related to AMR in agriculture by establishing interventions that will enhance the availability of data on AMU in animal, plants and other food products. This can be achieved by instituting collaborative efforts from all the responsible sectors to reduce the threat of AMR in agriculture across the region.

Limited availability of data is accompanied by the absence of a documented national surveillance system on AMU and AMR across the continent [27]. The dearth of information on AMU in animal production in Africa is partly contributed to by lacks of coordination or cooperation between national authorities and with private sector and livestock keepers, lack of tools and human resources and insufficient regulatory enforcements [25,26].

### 3.4. Antimicrobial Use in Aquaculture and Apiculture

Antimicrobials are also used in fish farms to prevent or treat bacterial and fungal infections, especially in hatcheries [30]. Other sources of antimicrobial contamination in fish result from the contamination of fish ponds by AMU in farming, animal husbandry, and the inappropriate disposal of domestic, hospital, and industrial wastes [9,31]. However, limited research has been conducted to determine the frequency of AMU, antimicrobial residuals and AMR in aquaculture settings in Africa. Antimicrobials have been used as growth promoters in the beekeeping industry, resulting in residues in honey [32]. In addition, AMU on fruit trees has been reported contaminate honey [33]. Antimicrobial contamination in honey results largely from their use to manage bacterial and parasitic diseases affecting bees [34].

### 3.5. Antimicrobial Resistance in Crop Production

Resistance levels and genes against antimicrobials used in crop protection have been reported against benomyl, carbendazim, carboxin, fenpropimorphe, mancozeb, prochloraz, tebucanazole, thiabendazole, and triadiemenol [35,36,37]. Moreover, several studies have indicated a strong link between the use of fungicides in agriculture and the clinical development of azole resistance *Aspergillus fumigatus* [38,39,40]. The AMU to control plant diseases has also been implicated with the dissemination of AMR in plants [41]. Furthermore, the use of manure and waste water for irrigation and contamination by handlers during harvesting, transportation and processing have been associated with developments and increases in AMR in plants, particularly vegetables and fruits [9,42]. Surprisingly, the AMU and mechanisms for AMR development in plants have not been well established [7].

The treatment of plant crops using triazoles is highly associated with exposure of all fungi in the field, and hence increases the risk of resistance development [35,36,37]. *Aspergillus fumigatus*, which is the most common fungal pathogen occurring in the environment, is frequently associated with chronic pulmonary and invasive aspergillosis, especially in immune-compromised patients [39]. Additionally, azole-sensitive and azole-resistant *A. fumigatus* has been reported to concomitantly infect patients with chronic pulmonary aspergillosis [38], posing more challenges in detection. Azole-resistant *A. fumigatus* (ARAf) due to TR34/L98H mutation in the cyp51A gene has been documented worldwide [39,43,44,45], including in Africa [46]. The TR34/L98H mutation is associated with an increased use of 14 alpha-demethylase inhibitor-based fungicides such as cyproconazole and propiconazole, which are commonly used in agricultural activities in Tanzania [20]. In Tanzania, azole resistance due to TR34/L98H mutations has been documented in clinical *A. fumigatus* of environmental origin [40].

### 3.6. Antimicrobial Resistance in Animal Production

AMR has been reported for *Escherichia coli*, Campylobacter, Salmonella, and Enterococcus species. Studies on AMR in Campylobacter species in Africa were available in 12 countries. The most commonly studied antimicrobials were tetracycline and quinolones (each in 9 countries). The most frequently targeted animals were poultry (12 countries) and cattle (5 countries) [47,48]. *Campylobacter* species including *C. jejuni* and *C. coli* are the organisms reported to be resistant to veterinary antimicrobials, probably due to imprudent use. Although resistance from a variety of animal species has been documented, very few studies involved molecular characterizations to document resistance genes. In a recent study in Nigeria, higher resistance levels of *Enterobactericeae* from samples of mutton, pork, beef and chicken to amoxycillin/clavulanic acid, streptomycin and perfloxacin have been reported [49] in *Campylobacter* spp. The proportion of Campylobacter MDR in poultry in Africa range from 11.1% to 100% (Table 1).

Resistant *Salmonella* spp. have been isolated from different animal species. Globally, it is the leading cause of foodborne zoonotic diseases [63]. *Salmonella* spp. and associated resistance genes have been detected from different animal species across Africa (Table 2). The resistance to ampicillin, sulphonamide, tetracycline, chloramphenicol, nalidixic acid, kanamycin and ciprofloxacin were mostly detected in the majority of the domestic animals tested. Studies in Kenya and Algeria documented ESBL producing *Salmonella* spp. [49,64,65,66].

*E. coli* has been reported to be highly resistant to several antimicrobials and is among the high-priority organisms [67]. Resistance in *E. coli* has been associated with mutations in the case of fluoroquinolones [68,69] or the production of enzymes such as extended spectrum beta-lactamases (ESBL) [69]. In *E. coli*, mobile genetic elements such as plasmids and transposons have largely been implicated in the dissemination of the resistance genes [50,70]. MDR *E. coli* has been detected in poultry in Algeria, Egypt, Ghana, Kenya, Nigeria, Tanzania, Zambia, and Zimbabwe [71,72,73,74,75,76,77,78,79]. In cattle, MDR *E. coli* has been detected in Egypt, Ethiopia, South Africa, Tunisia and Zambia [68,69,70,71]. In pigs, ducks, sheep, goats, rabbits, horses and camels, resistant *E. coli* have been reported from Angola, Cameroon, Nigeria, South Africa, Tunisia and Zimbabwe [72,73,74,75,76,77]. The level of resistance to common antibiotics such as ampicillin, tetracyclines, trimethoprim/sulfamethoxazole and the level of MDR *E. coli* have been reported to be very high. Of great concern is the detection of genes that confer resistance in animals, humans and the environment, or humans and the environment, or animals and the environment. This has been reported in Angola, Tanzania, South Africa, and Kenya [27,72,77,78,79]. The resistance genes detected were *bla*_CTX-M-15_, *bla*_TEM-1_, *bla*_OXA-1_, *aac(6*′*)-Ib-cr*, *bla*_CTX-M-15_, *bla*_CTX-M-15_, *bla*_TEM-1_, *bla*_OXA-1_, *aac(6*′*)-Ib-cr*, *strA*, *strB*, *aac(3)-IId*, *aadA1*, *qnrB1*, *qnrS1*, *sul1*, *sul2*, *dfrA14*, *dfrA17*, *dfrA1*, *dfrA18*, *dfrA30*, *dfrA5*, *dfrA7*, *tet(A)*, *tet(D)*, *aadA5*, *Tet(B)*, *sul1* and *tet(A).* In Africa, resistant *Enterococcus* spp. have been detected in a variety of food animals. *E. faecalis* and *E. faecium* were the most commonly studied resistant species (Table 3).

### 3.7. Antimicrobial Resistance in Aquaculture and Apiculture

An assessment of AMR in tilapia and catfish farms in Ghana found that out of 63 coliform bacteria tested, the majority were resistant to ampicillin (98.4%), cefuroxime (88.9%), tetracycline (66.7%), cefotaxime (52.4%), and co-trimoxazole (56.0%). Of the eight tested antimicrobials, gentamicin was the only effective drug (6.4%) [100]. A previous study on isolates from tilapia and catfish farms in Ghana reported that all *S. aureus* isolates exhibited 100% resistance to penicillin, ampicillin and flucloxacillin. All *E. coli* isolates were resistant to ampicillin, whereas the resistance to tetracycline, cotrimoxazole, cefuroxime and chloramphenicol ranged from 62.1% to 96.6%. Resistance of *Shigella* spp. isolates to ampicillin was 100%, whereas those to ciprofloxacin, tetracycline, cotrimoxazole, cefuroxime and chloramphenicol were at levels of 36.3% to 95.7%. *S. typhi* isolates exhibited 100%, 89.8% and 87.8% resistance to ampicillin, tetracycline and chloramphenicol, respectively. The resistance levels of *S. typhi* isolates to cotrimoxazole, cefuroxime, ciprofloxacin and gentamicin were 61.2%, 59.2%, 18.4% and 4.1%, respectively [101]. *S. aureus*, *E. coli*, *Shigella* spp. and *S. typhi* have been reported to show resistance against cotrimoxazole, cefuroxime, ciprofloxacin and gentamicin. MDR *S. aureus* isolates have also been reported from Ghana [101]. A study that investigated Gram-negative bacterial isolates belonging to diverse genera (including *Acinetobacter*, *Aeromonas*, *Bordetella*, *Chryseobacterium*, *Enterobacter*, *Myroides*, *Pseudomonas*, *Salmonella* and *Shewanella*) from tilapia farms found that all isolates (100%) were resistant to tetracycline [95]. The resistant gene determinants identified in decreasing order were *tet(A)*, *tet(E)*, *tet(B)*, *tet(C)*, *tet(D)* with prevalence rates of 40.9%, 31.8%, 29.6%, 25.0% and 6.8%, respectively.

A study that examined 100 isolates from tilapia sampled from fish markets in Ghana reported an overall prevalence of antibiotic residues of 7% [31]. Bacteria that were isolated from the fish samples were *Shigella sonnei* (10%), *Enterobacter cloacae* (7%), *Escherichia coli* (6%), *Salmonella typhi* (3%) and *Klebsiella pneumoniae* and *Proteus mirabilis* (2%). All bacteria isolated were susceptible to gentamicin and ciprofloxacin but resistant to ampicillin. Multi-drug resistance was identified in 86.7% of the isolates [31]. The study reported a further analysis on the prevalence of resistance to various antibiotics for *S. sonnei* isolates and observed that for eight of the ten drugs tested, the prevalence of AMR in increasing order was chloramphenicol/ceftriaxone/cefuroxime (25%), amikacin (33%), cefotaxime (42%), meropenem (67%), cotrimoxazole (83%) and ampicillin (100%).

A study in Nigeria examined 40 isolates of bacteria from fish farms and reported that all isolates were 100% resistant to ceftazidime, cefuroxime and augumentin. Resistance was also observed to cefixime (80%), gentamicin (73.3%) and nitrofurantoin (66.7%). The study further reported that 16.6% and 8.3% of the isolates were resistant to ciprofloxacin and ofloxacin, respectively [102]. Another study in catfish farms in Nigeria reported that out of the 20 *Salmonella hadar* isolates, 2 (10%) were resistant to neomycin, 1 (5%) was resistant to spectinomycin, and 9 (45%) were resistant to streptomycin, whereas 7 (35%) and 4 (20%) were resistant to sulfamethoxazole and trimethoprim, respectively. Of the two *Salmonella enterica* serovar, one each showed resistance to streptomycin and tetracycline. *Salmonella* spp. exhibited resistance to four antimicrobial agents, comprising ampicillin, sulfamethoxazole, tetracycline and trimethoprim. The overall patterns of AMR in this study showed that the majority of isolates were resistant to streptomycin (43.5%), sulfamethoxazole (34.8%), and trimethoprim (21.7%) [103]. A study in the Nile tilapia and African catfish farms in Uganda [104] detected *Aeromonas* spp. as being 100% resistant to penicillin and ampicillin and 23.2% resistant to cefotaxime. The study showed further that *Plesiomonas shigelloides* expressed 100% resistance to penicillin and oxacillin.

### 3.8. Drug Residues and Resistance in Food

Presences of drug residues and resistant bacteria in foods of animal origin have been reported by several authors across the region [2,105] (Table 4). AMR in meat products was reported from 14 countries across Africa, whereas AMR in vegetables and fruits was reported in 10 countries, most of them in West Africa, and AMR in eggs was only reported from Nigeria [11]. There are a few studies that have determined the level of antimicrobial residues in honey and AMR in Africa. A recent study on AMR in honey samples in Ghana reported an 11% prevalence of isolates of Listeria to both gentamicin and ciprofloxacin. About one quarter of the *Clostridium* spp. isolates showed resistance to either gentamicin or ciprofloxacin. Eighty-six percent of *Lactobacillus* spp. isolates were resistant to amikacin. Isolates of *Lactobacillus* spp. were resistant to roxithromycin (10%); ciprofloxacin (15%); azithromycin and gentamicin (5%). Isolates of *Staphylococcus* spp. recorded 6.7% resistance against azithromycin. All thirty isolates of *Salmonella* spp. were 100% resistant to ampicillin, cefuroxime, ceftriaxone and cefotaxime. Furthermore, all 30 isolates of *E. coli* were 100% resistant to ampicillin, cefuroxime, ceftriaxone, cefotaxime, chloramphenicol and ciprofloxacin [106].

### 3.9. AMU and AMR Policies in Africa

The tripartite collaboration of the World Health Organization (WHO), Food and Agriculture Organization (FAO) and World Organization for Animal Health (OIE) has provided international standards to guide the prevention and containment of AMR within human, animal, food and agriculture sectors. The Global Action Plan (GAP) on AMR was jointly developed by the three organizations. In the 68th World Health Assembly, the member states committed to develop One Health AMR National Action Plans (NAP). In May 2015, the OIE adopted the plan, which was further augmented by endorsement for the GAP by member states in September 2016. In addition, in May 2015, the OIE adopted Resolution No. 26 Combating AMR and Promoting Prudent AMU in Animals [128]; the FAO adopted a resolution on AMR in June 2015 [22]. The Codex Alimentarius Commission (CAC) adopted a code of practice to minimize and contain AMR and developed guidelines on the risk analysis of foodborne AMR [129]. These collaborative efforts have provided international standards guiding the prevention and containment of AMR within the human health and agriculture sectors. Following these international guidelines, a number of African countries have used the documents to prepare their AMR national action plans focusing on the One Health approach.

We observed that the majority of African countries had medicine policies emphasizing rational AMU in humans. Burkina Faso, Ghana, Nigeria, Ethiopia, Uganda, Kenya, Tanzania, South Africa and Zambia were found to have policies/guidelines addressing antimicrobial use in animal, food and agricultural systems using a One Health approach [130,131,132,133,134,135,136,137,138,139,140,141,142,143,144]. The policies require that all antibiotics to be used in humans must be dispensed upon the presentation of prescriptions. However, there is poor enforcement of these regulations in the animal and food sectors due to weak systems [145]. The policies observed in agricultural and food systems covered various areas such as AMR surveillance in animals, and the national regulatory framework for AMU in food production. They include policies limiting or restricting the emission of antimicrobials into the environment from farm waste; AMU surveillance in the animal sector; advertisements antimicrobials; antimicrobial in animal feed; and the promotion of antimicrobial stewardship programs or other initiatives focused on promoting responsible antimicrobial use and standard animal treatment guidelines.

Some countries such as Tanzania, Uganda, Ghana, Nigeria, Burkina Faso, Senegal and Zambia are at different stages of setting AMR/AMU surveillance systems. These systems include laboratory infrastructures, training of human resource, Standard Operating Procedures and data sharing platforms. These action plans address four objectives: (i) to improve the awareness and understanding of antimicrobial resistance; (ii) to strengthen knowledge through surveillance and research; (iii) to reduce the incidence of infection; and (iv) to optimize the use of antimicrobial agents. The plans also aim to develop the economic case for sustainable investment that takes account of the needs of the countries, and increases investment in new medicines, diagnostic tools, vaccines and other interventions [146].

Fourteen countries had national AMR action plans which clearly emphasized the One Health approach. However, except for Tanzania, no details were given regarding the implementation of these plans. In a recent study in Tanzania, despite some successes, the implementation of AMR NAP has faced a number of challenges. They include weakness in reporting and feedback mechanisms, accountability, transparency and sustainability of the plan [146]. It was observed that, in some countries, the AMR documents do not cover all necessary sectors. For instance, in Tanzania, despite the development of the plan involving several stakeholders, it was realized that the environmental sector was not involved [147]. Cross-sectoral coordination has been described to be a problem in low- and middle-income countries, including Thailand and Nepal [148,149]. In addition, the issues of AMU, especially in agricultural and food production systems, are not well covered. It was further noted that the monitoring of antimicrobial residues in food and the environment was poorly covered. Regarding antimicrobial stewardship (AMS), only Ghana, Kenya, South Africa, and Tanzania were found to have guidelines, one of the key strategies to overcome AMR [133,134,138,140]. However, the AMS guidelines in these countries focus only on the human health component, except for Kenya where guidelines for use in animal have been drafted. There is a need to further improve the documents to incorporate other key sectors.

In 2018, the Africa Centres for Disease Control and Prevention (Africa CDC) developed a framework for antimicrobial resistance control in Africa [150,151]. This guide outlines five key strategies to improve the diagnosis, treatment and collection of accurate AMR and AMU data and strengthen policies on AMR. The aim is to improve the surveillance of AMR organisms among humans and animals, delay emergence, limit transmission, and mitigate harm among patients infected with resistant pathogens [152]. The Africa CDC supports the development of operational plans by stakeholders based on the One Health approach.

## 4. Discussion

The findings of this review indicate that the AMU to control plant diseases as well as the use of manure and waste water for irrigation and contamination during harvesting, transportation and processing have been associated with developments and increases in AMR in crop production in Africa. On the other hand, antimicrobials such as tetracycline, streptomycin, benzimidazoles and triazoles that are frequently used to treat human and animal infections are also used to control plant diseases [16,17,18,19]. In Africa, cattle contribute to about half of the antimicrobial consumption in animal production. Tetracyclines account for about two-thirds of the antimicrobials used in animal production [23,24,25,26], although there are indications that the quantity of AMU in Africa has been decreasing during in recent years. AMR, including MDR, has been reported for several bacteria, including the WHO priority pathogens, mostly in cattle and poultry [47,48]. The levels of resistance to ampicillin, tetracyclines, trimethoprim/sulfamethoxazole in Africa are high; the genes that confer resistance in animals, humans and the environment have been reported in several countries in Africa [27,153,154,155].

This review found no study across Africa that reported data on antimicrobial consumption in plants, types of antimicrobial used, and the quantity used. Thus, it has been difficult to estimate AMU in the agricultural and other food production sectors. Lack of data on AMU in agriculture in Africa is caused by several reasons, including the weak surveillance of AMU in agriculture, lack of, or weakness in the enforcement of regulations, and the lack of guidelines and standards for surveillance of AMU and AMR. Similarly, although there is an increasing trend in AMU for animal production in Africa, data on the quantities used are limited [9,156]; this is likely attributed to the weakness of national surveillance systems and lack of coordination between national authorities and the private sector, lack of tools and human resources and insufficient regulatory enforcements [26,27].

Lack of surveillance of AMR in agriculture and food production systems makes it difficult to ascertain the trend of antimicrobial use and resistance in relation to policy enforcement. However, from the few research studies, the problem of antimicrobial resistance in agriculture/food production systems is high and increasing, indicating the need for policies/legislations with an enforcement plan. Furthermore, there are no surveillance data on antimicrobial use in animal and agriculture systems, making difficult to assess the impact of these policies on antimicrobial consumption. We also observed that there is lack of policies for antimicrobial uses in food and agricultural systems in Africa. In addition, the enforcement of the policies and legislations in these countries is a challenge due to a number of reasons, such as human resource and capacity to monitor the distribution and dispensing of antimicrobials. In addition, there is low awareness, competing priorities, costs, and a limited understanding of the relationship between resistance in animals, humans, and the environment. Key policies/regulations for addressing antimicrobial resistance and antimicrobial uses in agriculture/food production are needed in Africa. These should include policies and regulations on prescription practices, animal feed, surveillance of antimicrobial resistance and antimicrobial use, antimicrobial disposal, and strengthening the national drug regulatory authorities in the agriculture, human and animal health sectors. These policies should include the banning of over-the-counter sales and restrictions of non-therapeutic uses of antibiotics, while promoting alternatives to antibiotics such as probiotics, prebiotics and phytobiotics [157]. Already, Namibia has introduced a ban on the routine use of antibiotics in healthy animals [158].

## 5. Conclusions

The findings of this review indicate that, although there is limited information on the quantities of antimicrobials used in agriculture, aquaculture and apiculture, AMR has been reported for several pathogens and is increasing in Africa. Cattle and poultry production account for the largest consumption of antimicrobials in Africa. The levels of resistance to ampicillin, tetracyclines, trimethoprim/sulfamethoxazole in Africa are high; genes that confer resistance in animals, humans and the environment have been reported from several countries in Africa. Africa is lagging behind other parts of the world regarding the introduction of policies to combat antibiotic resistance, with fewer than one third of these countries having a National Action Plan for antimicrobial resistance. Due to the fact that many African countries have weaknesses in enforcing the policies and legislations in place, including the banning of over-the-counter antimicrobials, there is a need to establish/strengthen regulatory capacities to monitor AMU in agriculture and food production systems in Africa. It is equally important to promote alternative interventions to reduce the use of antimicrobials. Such interventions include good hygiene practices, biosecurity measures, and improved vaccination. In addition, probiotics, prebiotics and phytobiotics are likely to be the alternatives to antibiotics as growth promoters in food-producing animals. Addressing these issues will provide a sustained system to ensure policy enforcement. The One Health approach needs to be emphasized to facilitate the development of national, regional and global actions that will address AMR across all sectors.

## Figures and Tables

**Table 1:** Antimicrobial resistance in *Campylobacter* species by animal species and country.

Country	Animal	Antimicrobial Class	% MDR	Reference
Algeria	Poultry	Beta-lactams, Macrolides, Penicillin, Quinolones, Tetracycline	100	[49]
Botswana	Poultry	Beta-lactams, Fluoroquinolones, Tetracyclines	27	[48]
Côte d’Ivoire	Poultry	Aminoglycosides, Macrolides, Penicillin, Quinolones		[50]
Egypt	Cattle, buffalo, sheep, poultry	Aminoglycosides, Macrolides, Penicillin, Phenicols, Tetracyclines	-	[51]
Ethiopia	Poultry, cattle, pigs, sheep	Aminoglycosides, Cephalosporins, Macrolides, Penicillin, Sulphonamides, Tetracyclines	14.5	[52,53]
Ghana	Cattle, sheep, goat, pigs	Aminoglycosides, Beta-lactams, Macrolides, Phenicols, Quinolones, Sulphonamides Tetracycline	66.6	[54]
Kenya	Poultry	Quinolones, Tetracyclines	61.3	[47]
Kenya	Poultry	Aminoglycosides, Quinolones, Sulphonamides	79.22	[55]
Senegal	Poultry	Quinolones	11.1	[56]
South Africa	Poultry, pigs	Cephalosporins, Tetracyclines	43	[57]
South Africa	Poultry	Beta-lactams, Tetracycline	43	[58]
Tanzania	Cattle, goats, sheep, horses, camels	Macrolides, Quinolones, Lipopeptides	-	[59]
Tanzania	Pigs, cattle	Aminoglycosides, Beta-lactams, Furanes, Macrolides, Phenicols, Quinolones, Tetracyclines	56.8	[60]
Tunisia	Poultry	Macrolides, Quinolones, Phenicols, Tetracyclines	100	[61]
Zimbabwe	Poultry	Sulphonamides	-	[62]

**Table 2:** Resistance patterns of Salmonella species by animal species and country.

Country	Animal	Resistance Gene	Antimicrobial	References
Algeria	Poultry	*bla*_CTX-M-1_, *bla*_TEM_	Ciprofloxacin, Cefotaxime,	[67]
Algeria	Poultry		Ampicillin, Cephalothin, Tetracycline, Ofloxacin, Streptomycin, Enrofloxacin, Ciprofloxacin	[68]
Benin	Guinea fowl		Oxacillin, Sulfamethoxazole, Colistin	[69]
Burkina Faso	Cattle, poultry, pigs		Ampicillin, Chloramphenicol, Streptomycin, Sulphonamide, Trimethoprim	[50]
Burkina Faso	Poultry	*strA*, *strB*, *aadA1*, *bla*_TEM-1B_, *catA1*, *sul1*, *sul2*, *tetB*, *dfrA1*	Aminoglycoside, Beta-lactams, Phenicol, Sulphonamide, Tetracycline, Trimethoprim	[50]
Cameroon	Cattle, pigs		Tetracycline, Ampicillin, Amoxicillin, Doxycycline, Co-trimoxazole	[70]
Egypt	Poultry	*aadA1*, *aadA2*, *aadA*, *dfrA1*, *dfrA5*, *dfrA12*, *dfrA15*, *dfrA175*, *bla*_TEM-1_, *bla*_CMY-2_, *floR*, *tet(A)*	Ampicillin, Streptomycin, Spectinomycin, Kanamycin, Tetracycline, Chloramphenicol, Sulfamethoxazole–trimethoprim	[71]
Egypt	Poultry	*Bla*_TEM_, *tetA*, *tetC*, *sul1*, *sul3*, *cat1*, *floR*	Ampicillin, Tetracycline, Sulphamethoxazole, Chloramphenicol	[72]
Ethiopia	Poultry		Ampicillin, Chloramphenicol, Ciprofloxacin, Cefoxitin, Kanamycin, Nalidixic acid, Sulfamethoxazole–trimethoprim, Streptomycin, Tetracycline	[73,74]
Ethiopia	Cattle		Amoxicillin, Ampicillin, Chloramphenicol, Gentamycin, Kanamycin, Nitrofurantoin, Streptomycin, Sulfisoxazole, Tetracycline	[75,76,77,78]
Ethiopia	Cattle, pigs, poultry	*bla*_TEM_	Nalidixic acid, Ciprofloxacin	[79]
Ethiopia	Cattle, poultry		Gentamycin, Ciprofloxacin, Amoxicillin, Ampicillin, Streptomycin	[80]
Ghana	Poultry		Nalidixic acid, Tetracycline, Ciprofloxacin, Sulfamethoxazole, Trimethoprim, Ampicillin	[81]
Kenya	Pigs		Sulphonamide, Nalidixic acid, Tetracycline, Ampicillin, Erythromycin, Carbenicillin, Chloramphenicol, Gentamicin, Kanamycin, Spectinomycin, Sulfamethoxazole–trimethoprim, Streptomycin, Cephalothin, Ofloxacin, Ciprofloxacin, Norfloxacin	[65]
Kenya	Pig	*bla*_TEM_, *catA*1, *strA*, *tet(A)*	Ampicillin, Chloramphenicol, Streptomycin, Tetracycline	[66]
Nigeria	Poultry		Flumequine, Penicillin, Pefloxacin	[82]
Senegal	Poultry		Sulfamethoxazole–trimethoprim, Tetracycline, Trimethoprim, Streptomycin, Sulphonamide	[83]
South Africa	Cattle		Ampicillin, Cefotaxime, Cephalexin, Enrofloxacin, Erythromycin, Kanamycin, Oxytetracycline, Rifampicin	[84,85]
South Africa	Cattle, goats	*bla*_TEM_, *bla*_pse1_, *bla*_AmpC_	Tetracycline, Erythromycin	[86]
South Africa	Poultry		Ampicillin, Amoxicillin, Sulfamethoxazole–trimethoprim, Kanamycin, Gentamicin, Chloramphenicol, Erythromycin, Streptomycin, Tetracycline	[87]
Sudan	Poultry		Ciprofloxacin, Chloramphenicol, Tetracycline, Amikacin, Aztreonam, Ceftazidime	[88]
Sudan	Cattle, poultry, camels, sheep		Ampicillin, Chloramphenicol, Cephalexin, Furazolidone, Gentamicin, Kanamycin, Nalidixic acid, Streptomycin, Sulfamethoxazole–trimethoprim	[89,90]
Tunisia	Poultry	*sul1*, *sul3*, *strB*, *bla*_TEM-1_, *bla*_SHV_	Nalidixic acid, Ampicillin, Sulphonamide, Streptomycin	[91]
Uganda	Poultry	*bla*_TEM-1_, *cmlA*, *tetA*, *qnrS*, *sul1*, *dhfrI*, *dhfrVII*	Beta-lactams, Chloramphenicol, Tetracycline, Fluoroquinolones, Sulphonamide, Trimethoprim	[92]
Uganda	Pig		Sulfamethoxazole, Streptomycin, Trimethoprim, Chloramphenicol, Ampicillin, Tetracycline, Kanamycin	[59]
Zimbabwe	Poultry		Tetracycline, Ampicillin, Kanamycin	[93]

**Table 3:** *Enterococcus* virulent genes detected from different animals by antimicrobial agent and country.

Country	Animal	Enterococcus Species	Antimicrobial Agent	Virulent Gene Detected	Reference
Ethiopia	Cattle, poultry	*E. faecium*, *E. durans*, *E.hirea*, *E. faecalis*	Erythromycin, Ampicillin, Clindamycin, Amoxicillin, Cephalothin		[80]
Nigeria	Poultry, cattle	*E. faecium*, *E. gallinarum*, *E. faecalis*, *E. hirae*, *E. avium*, *E. raffinosus*, *E. durans*, *E. casseliflavus*, *E. mundtii*	Tetracycline, Gentamicin Erythromycin, Ampicillin	*asa1*, *gelE*, *cylA*, *tetK*, *tetL*, *tetM*, *tetO*, *ermB*	[94]
Nigeria	Poultry	*E. faecium*	Ampicillin, Aminoglycosides		[95]
Nigeria	Pigs	*E. faecalis*, *E. faecium*	Clindamycin, Penicillin, Erythromycin, Kanamycin, Gentamicin, Meropenem, Tetracycline	*asa1*, *gelE*, *Esp*	[96]
South Africa	Cattle	*E. faecalis*, *E. faecium*, *E. durans*, *E. avium*, *E. gallinarum*, *E. casseliflavus*, *E. mundtii*	Vancomycin, Linezolid, Penicillin, Erythromycin, Tetracycline, Ampicillin, Amoxicillin	*CylA*, *hyl*, *esp*, *gelE*, *asa1*	[97]
Tunisia	Poultry, cattle, sheep	*E. faecium*, *E. hirae*	Erythromycin, Tetracycline	*esp*, *ace*	[98]
Zambia	Cattle	*E.faecium*, *E. faecium*	Gentamicin, Amoxicillin, Ampicillin, Tetracycline		[99]

**Table 4:** Antimicrobial resistance reported from different food products.

Food Type	Country	Resistant Organisms	Drugs Resisted	References
Meat products (poultry, cattle, goat, sheep, etc.)	Côte d’Ivoire	*C. jejuni*, *C. coli*	Quinolones, Penicillin, Aminoglycosides	[50]
	Morocco	*E. coli*	Quinolones, Tetracycline, Penicillin, Clavam, Cephalosporin, Sulphonamides, Fluoroquinolones, Dihydrofolates, Aminoglycosides	[107]
	Algeria	*Salmonella* spp.	Sulphonamides, Quinolones, Aminoglycosides, Tetracycline	[108]
	Tunisia	*E. faecium*	Aminoglycosides, Ansamycins, Quinolones, Furanes	[109]
		*E. faecalis*	Macrolides, Tetracycline, Quinolones, Aminoglycosides, Phenicol	[96]
	Ethiopia	*Salmonella* spp.	Tetracycline, Sulphonamides, Dihydrofolates, Penicillin, Furanes, Cephalosporins	[110]
	Ghana	*Campylobacter* spp.	Penicillin, Quinolones, Aminoglycosides, Dihydrofolates, Tetracycline, Phenicol, Sulphonamides	[54]
		*E. coli*, *K. pneumoniae*	Quinolones, Aminoglycosides, Dihydrofolates, Sulphonamides	[111]
	Zambia	*E. coli*	Tetracycline, Sulphonamides, Penicillin, Dihydrofolates	[112]
	South Africa	*Salmonella* spp.	Aminoglycosides, Penicillin, Phenicol, Ansamycin, Cephalosporin, Tetracycline, Quinolones	[85]
	Gabon	*E. coli*, *S. aureus*	Quinolones, Penicillin	[113]
	Uganda	*Salmonella* spp.	Penicillin, Quinolones, Dihydrofolates, Sulphonamides	[114]
	Morocco	*S. enterica*	Tetracycline, Quinolones, Penicillin, Aminoglycosides, Clavam, phenicol, Dihydrofolates, Sulphonamides	[115]
	Cameroon	*Salmonella* spp.	Tetracycline, Aminoglycosides, Quinolones	[116]
	Senegal	*Salmonella* spp.	Dihydrofolates, Sulphonamides, Aminoglycosides, Tetracycline	[83]
	Kenya	*E. coli*	Tetracycline, Dihydrofolates, Sulphonamides, Penicillin, Aminoglycosides	[117]
	Tanzania	*E. coli*	Tetracycline, Penicillin	[118]
Eggs	Nigeria	*E. coli*, *Salmonella* spp.	Furanes, Sulphonamides, Penicillin, Dihydrofolates, Tetracycline	[119]
Vegetables and fruits		*E. coli*	Penicillin, Glycopeptide, Macrolides, Aminoglycosides, Ansamycins, Sulphonamides, Tetracycline, Dihydrofolates, Carbapenems	[11]
	Chad	*A. hydrophila*, *A. sobria*, *E. coli*, *Salmonella* spp., *S. aureus*	Penicillin, Clavam, Carbapenems, Quinolones, Phenocol, Cephalosporin, Aminoglycosides, Tetracycline, Glycopeptide, Sulphonamides, Dihydrofolates, Ansamycins	[120]
	Burkina Faso	*Salmonella* spp.	Aminoglycosides	[121]
	Uganda	*Salmonella* spp.	Cephalosporin	[12]
	Cameroon	*S. aureus*, *S. marcescens*	Aminoglycosides	[122]
	South Africa	*Salmonella* spp.	Phenicol, Aminoglycosides, Clavam, Penicillin, Sulphonamides, Dihydrofolates	[123]
	Ghana	*E. coli*, *S. enterica*	Quinolones, Penicillin, Macrolides	[124]
	Ghana	*Acinectobacter* spp., *E. coli*, *Aeromonas* spp., *Citrobacter* spp., *E. cloacae*, *S. aureus*, *K. pneumoniae*, *P. aureginosa*, *Salmonella* spp., *Serratia* spp., *Streptococcus* spp.	Penicillin, Cephalosporin, Phenocol, Tetracycline, Sulphonamides	[125]
	Tunisia	*E. coli*, *C. freundii*, *Enterobacter* spp., *K. pneumoniae*	Aminoglycosides, Macrolides, Phenicols, Penicillin, Quinolones, Sulphonamides, Tetracycline	[96]
		*E. faecium*, *E. hirae*, *E. faecalis*, *E. casseliflavus*	Quinolones, Macrolides, Tetracycline, Phenicols, Aminoglycosides, Glycopeptides	[42]
	Nigeria	*Enterobacter agglomerans*, *Proteus vulgaris*, *Klebsiella* spp., *Serratia liquefaciens*, *Staphylococcus* spp., *Bacillus* spp., *Pseudomonas fluorescens*.	Ampicillin, Cloxacillin, Augmentin, Erythromycin, Tetracycline, Cephalothin, Trimethoprim, Gentamicin, Ciprofloxacin, Ofloxacin	[126]
	Nigeria	*Bacillus* spp., *E. coli*, *Staphylococcus aureus*, *Pseudomonas* spp., *Klebsiella* spp., *Proteus* spp., *Corynebacterium*	Gentamycin, Ciprofloxacin, Ceftazidime, Cefotaxime, Perfloxacin, Cefuroxime, Ceftriaxone, Erythromycin, Vancomycin, Penicillin	[11]
	Algeria	*E. cloacae*, *K. pneumoniae*, *Acinetobacter*, *E. cloacae*, *Pseudomonas* spp., *C. murliniae*	Sulphonamides, Tetracycline, Aminoglycosides, Phenicols, Fluoroquinolones, Cephalosporin	[6]
	Benin	*E. coli*	Penicillin	[127]

## Data Availability

The data presented in this study are available on request from the corresponding author.
